# Bivalve Haemocyte Subpopulations: A Review

**DOI:** 10.3389/fimmu.2022.826255

**Published:** 2022-04-08

**Authors:** Nuria R. de la Ballina, Francesco Maresca, Asunción Cao, Antonio Villalba

**Affiliations:** ^1^ Centro de Investigacións Mariñas (CIMA), Consellería do Mar, Xunta de Galicia, Vilanova de Arousa, Spain; ^2^ MARE - Marine and Environmental Sciences Centre, Laboratório de Ciências do Mar, Universidade de Évora, Sines, Portugal; ^3^ Departamento de Ciencias de la Vida, Universidad de Alcalá, Alcalá de Henares, Spain; ^4^ Research Centre for Experimental Marine Biology and Biotechnology, Plentziako Itsas Estazioa (PIE), University of the Basque Country (UPV/EHU), Plentzia, Spain

**Keywords:** granulocyte, hyalinocyte, immune response, haematopoiesis, phagocytosis

## Abstract

Bivalve molluscs stand out for their ecological success and their key role in the functioning of aquatic ecosystems, while also constituting a very valuable commercial resource. Both ecological success and production of bivalves depend on their effective immune defence function, in which haemocytes play a central role acting as both the undertaker of the cellular immunity and supplier of the humoral immunity. Bivalves have different types of haemocytes, which perform different functions. Hence, identification of cell subpopulations and their functional characterisation in immune responses is essential to fully understand the immune system in bivalves. Nowadays, there is not a unified nomenclature that applies to all bivalves. Characterisation of bivalve haemocyte subpopulations is often combined with 1) other multiple parameter assays to determine differences between cell types in immune-related physiological activities, such as phagocytosis, oxidative stress and apoptosis; and 2) immune response to different stressors such as pathogens, temperature, acidification and pollution. This review summarises the major and most recent findings in classification and functional characterisation of the main haemocyte types of bivalve molluscs.

## 1 Introduction

Marine invertebrates constitute the largest group of macroscopic species in the sea (1). The phylum Mollusca is the second most diverse group of animals after Arthropods; among them, Bivalvia class is the second largest group of Mollusca that is worldwide distributed (2). They are often the major macrofauna on rocky substrates of littoral, shallow sub-littoral and deep-sea vents (3, 4). Bivalve molluscs are abundant in marine and freshwater ecosystems and perform important ecological functions. Bivalves have epifaunal or infaunal lifestyles and they are largely filter feeders that couple the water column and benthos. This filter-feeding habit adds greatly to their ecological significance in that bivalves are important calcium and carbon accumulators, they link primary producers (bacteria and phytoplankton) with higher organisms in aquatic food chains and are responsible for filtration of the water body (5, 6). Therefore, bivalve molluscs stand out for their fundamental role in the functioning of the aquatic ecosystems, impact nutrient cycling, create and modify habitat, and affect food webs (6). Moreover, they are used as environmental monitors because of the materials accumulated in their soft tissue and shells (7). Most bivalves, as sessile aquatic organisms, are exposed to an environment in continuous confrontation with pathogenic organisms and stressful conditions, such as dynamic variation in temperature, salinity and prolonged desiccation; so that throughout the evolution, these organisms have developed an array of effective strategies to protect themselves from the attacks of fast-evolving pathogens and environmental stresses, which has allowed them to obtain a high adaptation capacity to the different environments in which they live (8–11). In addition, both climate change and environmental pollution significantly affect the health of molluscs, potentially reducing the capacity of the bivalve immune system and increasing susceptibility to diseases (12–18). Altogether, the evolutionary ecological success of bivalves, showing a considerable resilience and occupying niches in a wide range of aquatic environments, is largely due to a robust, effective and multifaceted immune system which incorporates cellular and humoral components (8–11).

Hence, immune responses in bivalves and the processes that govern them, are important areas of active research. These immunological processes are centrally coordinated by a group of cells known as haemocytes which may act directly or in concert with humoral factors in the haemolymph to defend the animal against infection. Haemocytes constitute the cellular component of the haemolymph; they move through the circulatory system and migrate to other locations, such as the connective tissue and epithelia (19, 20). Among the important functions they perform in bivalves, haemocytes are best known for their primary role in phagocytosis, encapsulation and production of cytotoxic molecules, such as reactive oxygen species, antimicrobial peptides (AMPs) and secretion of inflammatory cytokines involved in pathogen killing and elimination (9, 19, 21–27). In addition to their role in host defence, bivalve haemocytes perform various important physiological functions, including nutrient digestion, transportation and distribution, wound healing, detoxification processes, shell mineralisation and excretion (25, 28, 29). The composition and dynamics of the bivalve haemocyte population, as well as the functional properties of circulating cells, reflect fairly objectively the general physiological and immunological status of bivalve molluscs and have an enormous potential for the study of physiological ecology (29–35). Due to the haemocyte major role in the immune system and homeostasis and the fact that few reports aimed to establish functional relationships between bivalve haemocytes subpopulations and immune response capacity, this review paper aims (i) to summarise current knowledge about bivalve haemocyte subpopulations classification and (ii) to point out functional differences between the main haemocyte types.

## 2 Bivalve Haemocytes Classification

**Figure 1:**
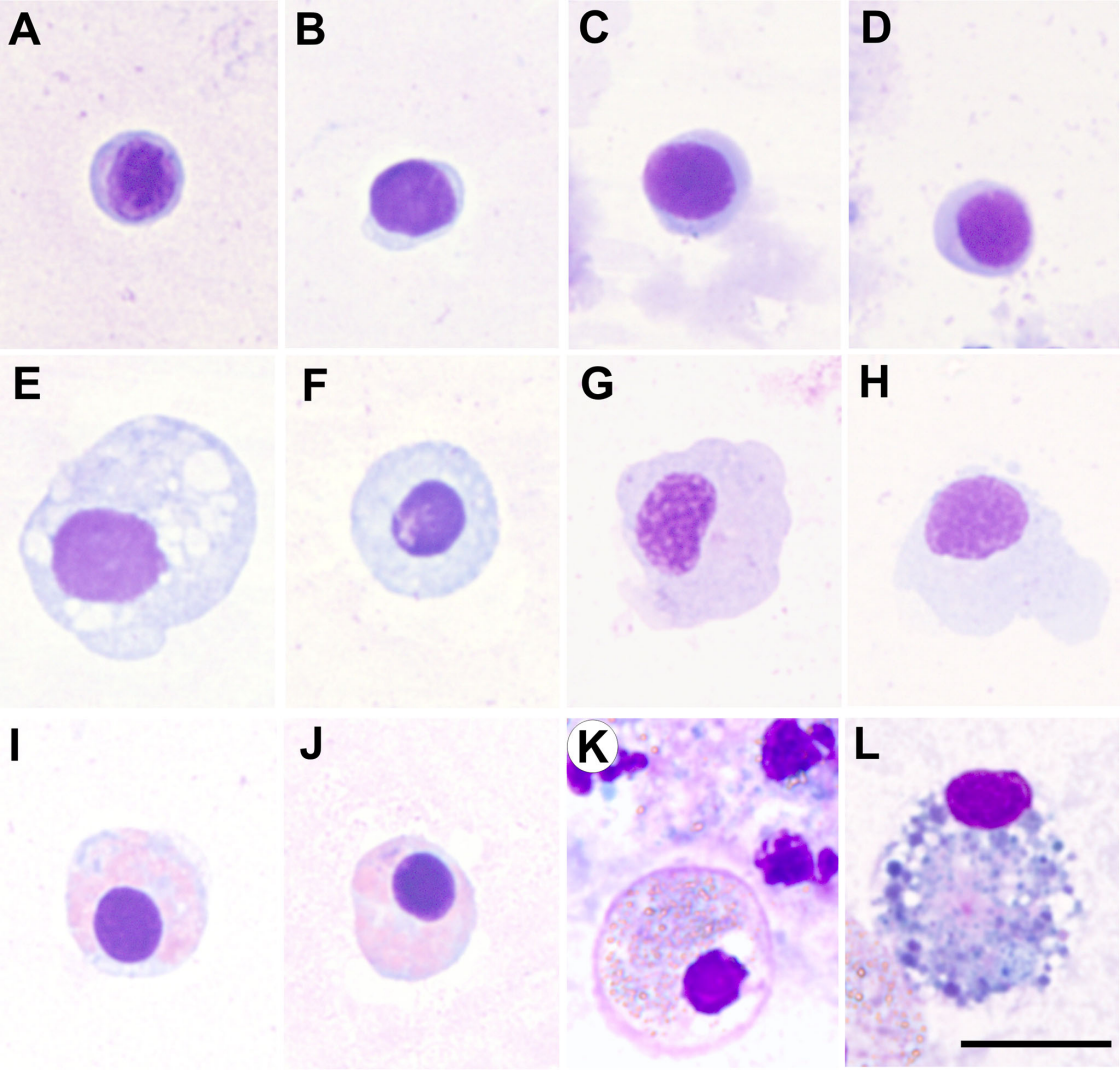
Micrographs of haemocyte types of various bivalve species, all them corresponding to haemolymph samples collected from the adductor muscle, cytocentrifuged onto slides and fixed and stained with the kit Hemacolor^®^ (Merck). **(A–D)** Blast-like cells of *Ruditapes decussatus*, *Ruditapes philippinarum*, *Aequipecten opercularis* and *Mimachlamys varia*, respectively. **(E–H)** Hyalinocytes of *R. decussatus*, *R. philippinarum*, *A. opercularis* and *M. varia*, respectively. **(I–K)** Eosinophilic granulocytes of *R. decussatus*, *R. philippinarum* and *Ostrea edulis*, respectively. **(L)** Basophilic granulocyte of *O. edulis*. Scale bar: 10 µm.

The classification of haemocyte populations or cell types in bivalve molluscs has been the subject of multiple studies since early 1970s. Research on haemocytes has been hindered by the lack of a consensus on their classification. A plethora of categories have been established on the basis of different parameters and techniques and, as a consequence, it is often difficult to compare results and draw general conclusions from the literature. Various criteria have been considered: cellular morphology (including ultrastructure), enzymatic cytochemistry, physicochemical features and cell population separation, and biological activities and functions. Numerous authors have focused their efforts on developing a classification of the different blood cell types present in bivalves. The researchers Cheng (1981) and Hine (1999) published two of the most important reviews on morphofunctional aspects of haemocytes of the Phylum Mollusca (19, 23). In the early studies, the haemocytes were characterised mainly by morphological and cytochemical criteria, such as the size, nucleus/cytoplasm (N/C) ratio, cytoplasmic complexity and enzyme content (36, 37). More recent trends for identifying haemolymph cell types are focused on flow cytometry, tool that allows determining the size and granularity of haemocytes (38). Most studies have classified bivalve haemocytes into two main groups: granulocytes, cells with granules in the cytoplasm and typically a low N/C ratio, and hyalinocytes (or agranulocytes), cells containing few or no granules within the cytoplasm and a higher N/C ratio (19, 21, 36, 39) (**Figure 1**). **Table 1** brings together the different studies focused on categorising the haemocytes subpopulations in bivalves. Both cell types, granulocytes and hyalinocytes (or agranulocytes), have been found in clams, razor shells, scallops, cockles, mussels, and oysters. In many of these species, granulocytes contain hydrolytic and oxidative enzymes and may be further subclassified into different categories based on granular affinity to specific dyes, such as acidophilic/eosinophilic, basophilic and neutrophilic granulocytes; or different subtypes according to the size and granularity (23, 60, 108, 181). Cheng suggested that the occurrence of various types of granules might be related to differentiation and maturation processes; specifically, basophilic granules were hypothesised to be immature granules which mature and become acidophilic (19). Recently, up to fourteen types of granules were identified in the clam *Ruditapes philippinarum* granulocytes (75) and up to twelve haemocyte subpopulations were identified in the oyster *Crassostrea hongkongensis*, the latter based on transcriptomic profile of single cell RNA-seq data (182). One of the most recent morphology classification of bivalve haemocytes was made with a new computational approach that combines fractal formalism with linear methods of image analysis (183, 184); however, results are not easily comparable with classic haemocyte classification.

**Table 1:** Haemocyte subpopulations in bivalve mollusc species.

**SPECIES**	**HAEMOCYTE TYPES**						**REFERENCES**
	**G**	**sG**	**H**	**sH**	**B**	**Other cells**	
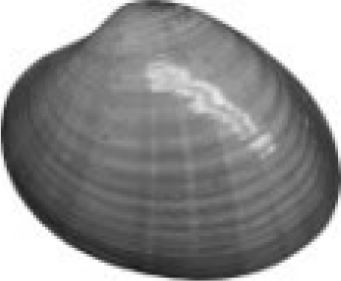	**CLAMS** (32 species)						
**Family Astartidae**							
*Astarte borealis* (*Mactra veneriformis)	G ^P^	sG				A_G_ (Small & Large)	(40) ^P^
**Family Cardiidae**							
*Tridacna crocea*	G		H			Morula-like	(41)
*Tridacna maxima*	2types						(42)
**Family Cyrenidae**							
*Corbicula japonica*	2types		H	sH			(43)
*Villorita cyprinoides*	G						(44)
**Family Hiatellidae**							
*Panopea globosa*	G ${}_{\text{O}\text{X}}^{\text{P}}$		H	sH			(45) ${}_{\text{O}\text{X}}^{\text{P}}$
**Family Laternulidae**							
*Laternula elliptica*	G					A_G_	(46)
**Family Mactrinae**							
*Mactra antiquata* (*Coelomactra antiquata)	G	sG	H				(47)
*Spisula solidissima*	G					A_G_	(18)
**Family Mesodesmatidae**							
*Amarilladesma mactroides* (*Mesodesma mactroides)	G		H				(48)
*Paphies ventricosa*	G		H				(49)
**Family Myidae**							
***Mya arenaria***	G					A_G_	(50)
	G		H				(51)
**Family Psammobiidae**							
*Hiatula diphos* (*Sanguinolaria diphos)	G	sG	H				(47)
**Family Semelidae**							
*Scrobicularia plana*	2types					A_G_	(52)
**Family Solecurtidae**							
*Tagelus plebeius*	G		H				(53)
**Family Tellinidae**							
*Tellinimactra edentula* (*Macoma edentula)	G		H				(54)
**Family Veneridae**							
*Callista chione*	2types		2types				(55)
*Chamelea gallina*	G		H				(56)
*Leukoma thaca* (*Protothaca thaca)	G		H				(57)
*Macrocallista nimbosa*						Haemocyte	(58)
***Meretrix lusoria***	G	sG	H			Fibrocyte	(59)
	G	sG	H		B		(60)
	G ^P^	sG	H				(61) ^P^
*Meretrix meretrix*	G	sG				A_G_, Lymphoid cell	(62)
*Meretrix petechialis*	G				B	A_G_, Degranulated cell	(63)
*Mercenaria campechiensis*	G					A_G_	(64)
***Mercenaria mercenaria***	G ^P^		H			Fibrocyte	(65–67) ^P^
		2types				B	A_G_	(68)
***Paratapes undulatus*** (*Paphia undulata)	G	sG	H				(47)
	2types		2types				(69)
*Protapes gallus* (*Paphia malabarica)	G					A_G_	(70)
***Ruditapes decussatus***	3types ^P^		H				(71, 72) ^P^
	G _ox_		H			Intermediate cell	(73) _ox_
***Ruditapes philippinarum*** (*Tapes philippinarum)	G		H		B	Serous cell	(74)
	G ${}_{\text{O}\text{X}}^{\text{P}}$		H		B		(29, 75) ${}_{\text{O}\text{X}}^{\text{P}}$
*Sunetta scripta*	G					A_G_	(44)
**Family Vesicomyidae**							
*Abyssogena phaseoliformis*	2types					Erythrocyte	(76)
*Phreagena okutanii*	2types					Erythrocyte	
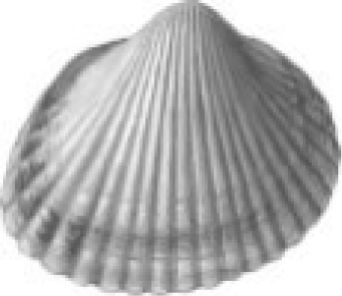	**COCKLES** (2 species)						
**Family Cardiidae**								
***Cerastoderma edule***	G		H			Type III cell	(77)	
	2types					A_G_, Type III eosynophil	(78)
*Cerastoderma glaucum*	2types ^P^		2types				(79) ^P^
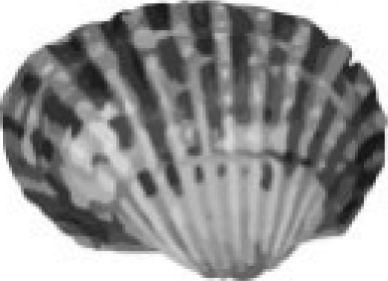	**ARK CLAMS** (9 species)						
**Family Arcidae**							
*Anadara antiquata*						Red cells, White cells, Thrombus cells	(80)
***Anadara broughtonii*** (*Scapharca broughtonii)						Red cells, White cells, Platelets	(81)
	G ${}_{\text{O}\text{X}}^{\text{P}}$		H		B	Erythrocyte I & II	(82) ${}_{\text{O}\text{X}}^{\text{P}}$
***Anadara inaequivalvis*** (*Scapharca inaequivalvis)	3types					Erythrocyte	(83)
	3types					A_G_, Fibrocyte, Monocyte, Platelets	(84)
***Anadara kagoshimensis*** (*Scapharca subcrenata)	G		H			Erythrocyte	(85)
						Amebocyte, Erythrocyte, Intermediate cell	(86)
	G ${}_{\text{O}\text{X}}^{\text{P}}$		H		B	Erythrocyte I & II	(82) ${}_{\text{O}\text{X}}^{\text{P}}$
*Anadara trapezia*						Amebocyte, Erythrocyte	(87)
*Lunarca ovalis* (*Anadara ovalis)	G					A_G_, Erythrocyte	(64)
*Senilia senilis* (*Anadara senilis)						Red cells, White cells, Platelets	(88)
***Tegillarca granosa***	2types		H				(89)
	G ${}_{\text{O}\text{X}}^{\text{P}}$		H		B	Erythrocyte I & II	(82) ${}_{\text{O}\text{X}}^{\text{P}}$
*Tegillarca rhombea*	G					Red cells, A_G_, Platelets	(90)
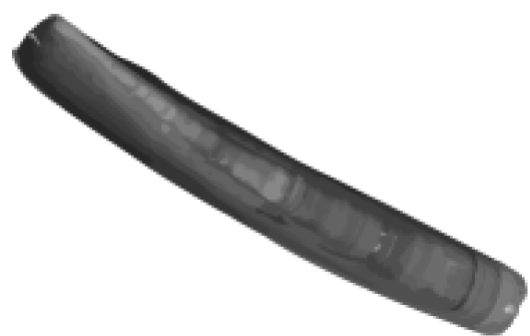	**RAZOR SHELLS OR RAZOR CLAMS** (3 species)						
**Family Pharidae**							
Ensis leei (*Ensis directus)	G	sG	H	sH		Vesicular cell	(91)
*Ensis siliqua*	2types					A_G_	(78)
*Sinonovacula constricta*	G ^P^		H			SemiG	(92) ^P^
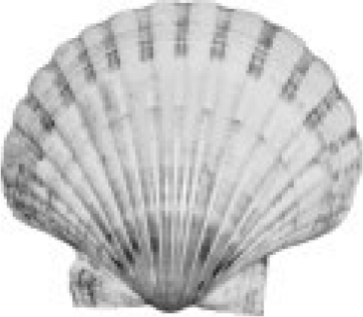	**SCALLOPS** (4 species)						
**Family Pectinidae**							
*Argopecten irradians*	G ^P^	sG	H	sH			(93) ^P^
*Argopecten purpuratus*	G		H				(94)
***Azumapecten farreri*** (*Chlamys farreri)	G		H				(95)
	2 types ^P^	sG	H	sH			(96) ^P^
***Nodipecten subnodosus***			H		B		(97)
			H		B	SemiG	(98)
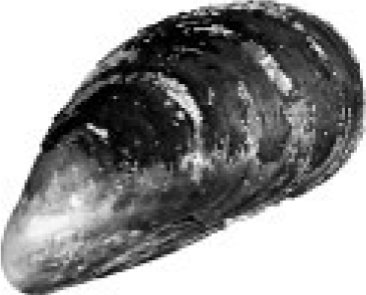	**MARINE MUSSELS** (21 species)						
**Family Mytilidae**							
*Aulacomya atra* (*Aulacomya ater)	G		H				(99)
*Bathymodiolus azoricus*	G ^P^		H		B		(100, 101) ^P^
*Bathymodiolus japonicus*	2types					A_G_	(102)
*Gigantidas platifrons* (*Bathymodiolus platifrons")	2types					A_G_	
*Bathymodiolus septemdierum*	2types					A_G_	
*Brachidontes pharaonis*	G	sG	H				(103)
***Modiolus kurilensis***	G				B	A_G_, SemiG	(104)
	G				B	A_G_	(35)
*Modiolus modiolus*	G		H			SemiG	(105)
*Mytella strigata* (*Mytella falcata)	G					A_G_	(106)
*Mytilisepta virgata*	G ^P^		H		B		(107) ^P^
*Mytilus californianus*	G					A_G_ (2types)	(108)
*Mytilus chilensis*	G ^P^		H				(109) ^P^
***Mytilus edulis***	G ^P^					Lymphoid cell, Macrophage	(110) ^P^
	2 types					A_G_	(111)
	G		H			Basophil	(112)
***Mytilus galloprovincialis***	3types ^P^		H				(113) ^P^
	G _ox_		H			SemiG (Small & Large)	(114) _ox_
	G		H				(115)
*Mytilus platensis* (*Mytilus edulis desolationis)	G		H				(99)
*Mytilus trossulus*	G					A_G_	(116)
*Mytilus unguiculatus* (*Mytilus coruscus)	G ${}_{\text{O}\text{X}}^{\text{P}}$		H		B		(117) ${}_{\text{O}\text{X}}^{\text{P}}$
***Perna canaliculus***	2types		H				(118)
	G ${}_{\text{O}\text{X}}^{\text{P}}$		H		B		(119) ${}_{\text{O}\text{X}}^{\text{P}}$
*****Perna perna*****	G ^P^		H				(120) ^P^
	G		H		B	SemiG	(121)
***Perna viridis***	G ${}_{\text{O}\text{X}}^{\text{P}}$		H				(122) ${}_{\text{O}\text{X}}^{\text{P}}$
	G		H		B	SemiG (Small & Large)	(123)
*Xenostrobus securis*	G ${}_{\text{O}\text{X}}^{\text{P}}$		H		B		(124) ${}_{\text{O}\text{X}}^{\text{P}}$
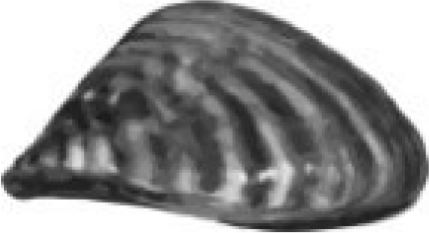	**FRESHWATER MUSSELS** (18 species)						
**Family Dreissenidae**							
*Dreissena bugensis*	G		H		B		(125)
***Dreissena polymorpha***	G		H				(126)	
	G		H		B		127)	
**Family Hyriidae**							
*Diplodon chilensis*	G ^P^		H		B		(128) ^P^
**Family Mycetopodidae**							
*Anodontites trapesiali*	G		H	sH	B		(129)
**Family Unionidae**							
*Amblema plicata*	2types ^P^					A_G_ (Small & Large)	(130, 131) ^P^
*Anodonta anatina*	G		H				(132)
***Anodonta cygnea***	G		H				(133)
	2types		H		B	Vesicular cell	(134)
*Anodonta woodiana*	G		H			A_G_, Lymphoidocyte	(135)
*Cristaria plicata*	G	sG	H			Lymphoid cell	(136)
*Elliptio complanata*	G		H				(137)
*Hyriopsis bialata*	G					A_G_ (Small & Large)	(138)
*Lamellidens marginalis*	G ${}_{\text{O}\text{X}}^{\text{P}}$		H		B	A_G_, Asterocyte	(139, 140) ${}_{\text{O}\text{X}}^{\text{P}}$
*Lampsilis rafinesqueana*	G		H				(141)
*Quadrula* sp.	2types ^P^					A_G_ (Small & Large)	(131, 142) ^P^
*Solenaia oleivora*	G	sG	H			Lymphoid cell	(143)
*Sinohyriopsis schlegelii* (*Hyriopsis schlegeli)	G		H			Serous cell, Lymphoid cell, Spindly cell, Thrombocyte	(144)
***Sinohyriopsis cumingii*** (*Hyriopsis cumingii)	G ^P^		H				(145) ^P^
	G		H			Lymphocyte, Spindly cell, Thrombocyte	(146)
*Unio pictorum*	G		H				(147)
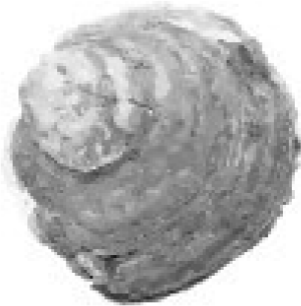	**OYSTERS** (17 species)						
**Family Gryphaeidae**							
*Hyotissa hyotis*	G ${}_{\text{O}\text{X}}^{\text{P}}$		H		B		(148) ${}_{\text{O}\text{X}}^{\text{P}}$
**Family Ostreidae**							
*Alectryonella plicatula* (*Ostrea plicatula)	2types	2types	2types				(69)
*Crassostrea brasiliana* (*Crassostrea gasar)	G		H		B		(149)
***Crassostrea ariakensis***	G		H	sH			(150)
	G ${}_{\text{O}\text{X}}^{\text{P}}$		H		B		(151) ${}_{\text{O}\text{X}}^{\text{P}}$
*Crassostrea corteziensis*	G		H				(152)
***Crassostrea gigas***	G		H	sH			(153)
	G		H			Small A_G_	(154) _ox_
	G		H		B		(155)
	G ${}_{\text{O}\text{X}}^{\text{P}}$					A_G_, SemiG	(156) ${}_{\text{O}\text{X}}^{\text{P}}$
***Crassostrea hongkongensis***	G ${}_{\text{O}\text{X}}^{\text{P}}$		H				(157) ${}_{\text{O}\text{X}}^{\text{P}}$
	G					A_G_, SemiG	(158)
*Crassostrea madrasensis*	G		H			SemiG	(159)
*Crassostrea nippona*	G ${}_{\text{O}\text{X}}^{\text{P}}$		H		B		(160) ${}_{\text{O}\text{X}}^{\text{P}}$
***Crassostrea rhizophorae***	G		H				(161)
	G				B	A_G_	(162)
*Crassostrea plicatula*	2types ^P^	2types	2types				(163) ^P^
***Crassostrea virginica***	G		H			Fibrocyte	(164)
	G	sG	H				(165)
	G	sG	H	sH			(166)
	G ${}_{\text{O}\text{X}}^{\text{P}}$		H			Intermediate cells	(167) ${}_{\text{O}\text{X}}^{\text{P}}$
***Ostrea chilensis*** (*Tiostrea chilensis)	2types		H			Serous cell	(168)
	2types ${}_{\text{O}\text{X}}^{\text{P}}$		H				(169) ${}_{\text{O}\text{X}}^{\text{P}}$
*Ostrea circumpicta*	G ${}_{\text{O}\text{X}}^{\text{P}}$		H		B		(148) ${}_{\text{O}\text{X}}^{\text{P}}$
***Ostrea edulis***	G ^P^		H	sH			(153, 170) ^P^
	2types	sG	H	sH			(171)
*Saccostrea cuccullata*	G		H				(172)
***Saccostrea glomerata***	5types ${}_{\text{O}\text{X}}^{\text{P}}$		H		B		(173) ${}_{\text{O}\text{X}}^{\text{P}}$
	G		H			Small A_G_	(174)
*Saccostrea kegaki*	G ${}_{\text{O}\text{X}}^{\text{P}}$		H		B		(148) ${}_{\text{O}\text{X}}^{\text{P}}$
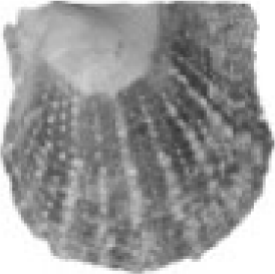	**PEARL OYSTERS** (3 species)						
**Family Margaritidae**							
*Pinctada imbricata*	G ^P^	sG	H		B	Serous cell	(175) ^P^
*Pinctada fucata*	G		H	sH			(176)
*Pinctada margaritifera*	G		H	sH			(177)
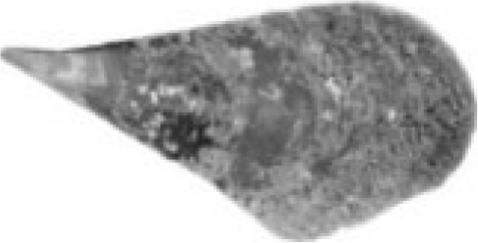	**PEN SHELLS and WING SHELLS** (2 species)						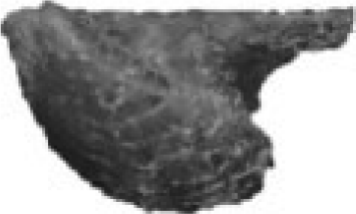
**Family Pinnidae**							
***Pinna nobilis***	3types		2types	sH			(178)
	3types		H				(179)
**Family Pteriidae**							
*Pteria hirundo*	G		H	sH	B		(180)

^p^: Species in which granulocytes show higher phagocytosis capacity than hyalinocytes.

_ox_: Species in which granulocytes show higher oxidative activity through ROS production than hyalinocytes.

Species in **bold**: haemocyte subpopulations vary according to different authors for the same species. *synonym.

G, granulocyte; sG, small granulocyte; H, hyalinocyte; sH, small hyalinocyte; B, blast-like cell; A_G_, agranulocyte; SemiG, semigranulocyte.

The relative abundance of each cell type in the haemolymph of bivalve molluscs is variable, being affected by seasonal changes, temperature, size, sex, maturity, food availability and inter-individual variability (43, 70, 185–190). In bivalves, granulocytes are generally considered the most abundant cell type (191). Nevertheless, in some studies a greater number of hyalinocytes (or agranulocytes) have been observed, such as in the marine mussels *Perna perna* (120), *Perna canaliculus* (119) and *Mytilus chilensis* (109); the freshwater mussels *Dreissena polymorpha* (127) and *Diplodon chilensis* (128); the clams *Chamelea gallina* (56), *Meretrix meretrix* (62) and *Ruditapes decussatus* (192); the lantern clam *Laternula elliptica* (46); the boreal tridonta *Astarte borealis* (40); the geoduck clam *Panopea globosa* (45); the scallop *Argopecten irradians* (93); the oysters *Crassostrea brasiliana* (149), *Crassostrea gigas* (155), *C. hongkongensis* (157), *Crassostrea rhizophorae* (162), *Saccostrea glomerata* (173), *Saccostrea kegaki, Ostrea circumpicta* (148), *Ostrea edulis* (171) and *Hyotissa hyotis* (148); the wing-shell *Pteria hirundo* (180); and the pearl oysters *Pinctada fucata* (176) and *Pinctada margaritifera* (177).

Although in almost all the studied species it is possible refer to the two cell types as hyalinocytes (agranulocytes) and granulocytes, there are some exceptions and not all haemocyte types occur in each bivalve species. In the clam *Macrocallista nimbosa*, flow cytometrically characterised haemocytes appeared as an unique population, both in terms of morphology and intracellular parameters (58). Some authors disagree with the existence of granular cells in pectinids (23, 39, 193); however, different studies have found granulocytes in the haemolymph of some scallop species (93–96), thus a sole rule would not apply for all the scallop species. In the scallop *Nodipecten subnodosus*, classification into main types of haemocytes (hyalinocyte and granulocyte) was not deemed totally correct, considering a haemocyte subpopulation as semi-granular cells (98). Most of ark clams, species of the family Arcidae, also known as “blood clams”, have erythrocytes, haemocytes containing the respiratory pigment haemoglobin, a rare occurrence in invertebrates (194, 195). Although generally shallow-sea veneroid clams have no erythrocytes in the haemolymph, erythrocytes were found as the most abundant cells in the species *Phreagena okutanii* and *Abyssogena phaseoliformis* (76). A particular type of haemocyte, without cytoplasm granules, with a large vacuole occupying most of the cytoplasm and peripheral flattened nucleus, has been observed in the common edible cockle *Cerastoderma edulis*, reported as type III (77, 78), and in the lagoon cockle *Cerastoderma glaucum*, reported as acidophil granulocyte (79).

Another haemocyte type frequently found in bivalves is the blast-like cell (**Figure 1**, **Table 1**), this cell type exhibiting high N/C ratio and small size and apparent low levels in biological activities (hydrolytic enzymes, oxidative activity, phagocytosis, number of lysosomes) (74, 127, 151). Blast-like cells designation remains variable and different authors use different denominations to identify this cell type: lymphoid haemocytes (62, 110, 136), basophils (112), haemoblast-like (104, 123, 162, 173) or small hyalinocytes (170), referring to the same haemocyte type (127). As discussed below, blast-like cells are considered haemocyte precursors according to their morphometric parameters and low organelle content (19, 23, 162).

There is not a unified classification system for haemocytes in bivalve molluscs, thus far. On the contrary, the haemocyte types have been reported differentially between species. Even for the same species, different haemocyte classification has been proposed, as shown in **Table 1** (species in bold). This diversity may be in part due to some true differences, but also resulting from the use of different classification criteria or experimental procedures, endogenous and exogenous factors, like age, pollution or the high inter-individual variability (71, 77, 79, 112, 196, 197). Additionally, different nomenclatures adopted by various researchers due to lack of biological markers for specific cell lineages or maturation stages contribute to the problem (198). Moreover, the process of haematopoiesis in bivalves is not completely clear yet, therefore, the lack of evidence on the origin of haemocytes hampers classification because no correspondence between haemocyte subtypes and ontogeny can be made (162).

## 3 Bivalve Haematopoiesis

Haematopoiesis is a crucial and vital process for homeostasis and immune response against infection in invertebrate animals and therefore for survival (198). Various theories have been proposed to elucidate the lineage of haemocytes in bivalves. Cheng (1981) and Auffret (1988) propose two types of initial cell precursors capable of differentiating into granulocytes and hyalinocytes (19, 39). Alternatively, a model with a single precursor cell type giving rise to hyalinocytes that later mature into granulocytes was suggested first by Mix (199), and then by Hine (23). In the case of the mussel *M. galloprovincialis* haemolymph, the occurrence of only one haemocyte type represented by two different ageing-related stages has been proposed; specifically, hyalinocytes in a proliferative stage which mature to become granulocytes (200), consistently with the Mix’s one-cell-type model (199). In the clam *R. philippinarum*, a single population of precursor cells lacking granules in their cytoplasm called blast-like cell has been described; these cells were mitotic haemocytes positive for CD34, a transmembrane glycoprotein characteristic of haematopoietic mammalian cells (74, 201), thus supporting the hypothesis proposed by Hine (23). Unlike granulocytes and hyalinocytes, precursor cells do not contribute to immune response mechanisms such as phagocytosis or encapsulation, and they also lack common intracellular enzyme systems associated with host defence. The presence of few cytoplasmic organelles and low enzyme activity suggests that these precursor cells are immature haemocytes (23, 74) leaving open the possibility that these cells act as stem cells from which derive the two classes of mature haemocytes. In many invertebrates, multiple types of haemocytes appear to derive from the differentiation of stem cells that have a morphology very similar to the cells of *S. glomerata* that are considered haemocyte precursors (173, 202, 203). Those potential haemocyte precursors, also called blast-like cells, have been widely found in clams, ark clams, scallops, marine mussels, freshwater mussels, oysters, pearl oysters and wing-shells as shown in **Table 1** (column B). Rebelo *et al.* (162) proposed another hypothesis in which the different haemocyte types derive from the same cell type that matures first without granules (hyalinocyte), later produces granules (granulocyte) and finally, eventually, they can lose the granules being an agranular cell again. Thus, different haemocyte subpopulations have been indicated as different stages of one cell type only; theory similar to that proposed by Ottaviani (200). The hypothesis by Rebelo et al. is based on observations made in *C. rhizophorae* (162) and later in *C. virginica* (204). A recent study identified different stages of granulocytes in oysters *C. hongkongensis*, which led authors to propose that several differentiation states may exist within one cell type in the haemocyte formation process (182). Despite various theories, the detailed characterisation of larval and adult haematopoiesis in bivalves will only be possible by sequencing mollusc genomes and identifying the full set of transcription factors and biomarkers that regulate haematopoiesis (198, 205, 206).

Even being an essential process in bivalve immunity, there is no clear haematopoietic organ or cell precursor, although the generally accepted belief is that haemocytes can originate from connective tissue and/or mantle (19, 199, 207). In spite of some haemocytes may mature before entering the circulation (29, 79, 123, 162), evidence is accumulating that mitosis may also occur after haemocyte release into the haemolymph (201). In the oyster *C. gigas*, important vertebrate embryonic haematopoiesis transcription factors have been found expressed during ontogeny (208, 209), which were observed only in cells attached to the blood vessel endothelium, leading the authors to hypothesise that haematopoietic cells could derive from the vessel and/or artery endothelial cells (209). It has been also proposed that haemocytes can be differentiated from a population of adult somatic cells residing in an irregularly folded structure in the gill of the adult oyster *C. gigas* (210), suggesting that gills can potentially act as the haematopoietic organ in oysters (211), which bears out the early proposition by Cuénot (212) that bivalve haemocytes originate in the gills. To this extent, a very recent study suggests that stem cells firstly divide and differentiate into pro- haemocytes in gills (213). Also in the oyster *C. gigas*, some haematopoietic relative genes have been found up-regulated after bacterial infection (214); this evidence also supports the hypothesis that in bivalves the proliferation of circulating haemocytes may occur as a consequence of an immune challenge (215). In addition, in the same species some transcription factors were identified in granulocyte-specific genes with strong potentials in regulation of haematopoiesis (216). In the scallop *Azumapecten farreri*, several molecules associated with the proliferation and differentiation of pro-haemocytes have been identified and characterised from the circulating haemocytes (217). These molecular studies are providing evidence that precursors of fully differentiated haemocytes can occur in the circulatory system of bivalves, where presumably they continue to mature (218). In oysters *C. gigas*, a conserved haematopoietic transcription factor has been found expressed in important immune organs, such as gills, mantle and haemocytes (219). Moreover, recently, another transcription factor involved in haematopoiesis has been found highly expressed in gills and haemocytes, with higher abundance in semigranulocytes and agranulocytes (220). This fact, added to that a definitive haematopoietic site common to all bivalves has yet to be identified, lead to the possibility that adult bivalves do not produce haemocyte precursors or mature haemocytes from a centralised organ, as occurs in other molluscan taxa; multiple or ubiquitous sites of haematopoiesis may exist, comprising a system in which stem-like cells receive determining signals from neighbouring specialised cells or tissues (206). Therefore, a bivalve haematopoietic organ is not the norm; haemocytes may instead be formed in various ways (216). Spontaneous mitosis of haemocytes increases during circulation in haemolymph vessels, sinuses, and soft tissues (19, 198), which raises the possibility of observing plasticity during various stages of haemocyte maturation (162, 199, 200).

## 4 Functional Differences Between Hyalinocytes and Granulocytes

For the purposes of this review, we will refer to the two haemolymph cell types as hyalinocytes (agranulocytes) and granulocytes, while acknowledging that these may be different life stages of the same cell type in some species. In spite that Cheng (36) suggested that different haemocyte types perform distinct functions in the 1980s, the involvement of the different haemocyte subpopulations in immune functions is still far from well understood. Initially, the distinction between hyalinocytes and granulocytes was made based on morphological parameters, years later it has been confirmed that there are also functional differences between both haemolymph cell types after analysis of metabolic and functional parameters (122, 221). Recently, research efforts are focusing on revealing dissimilar immune functions among marine invertebrate haemocyte subpopulations, for example in ascidians (222); crustaceans as shrimps (223–228), lobsters (229) and crabs (230); gastropod molluscs as sea snails (231–234), abalones (235) and sea hares (236); and bivalve molluscs as oysters (182, 216, 237, 238). This review focuses on the major findings in functional differences between the main bivalve haemocyte types.

### 4.1 Immune Parameters

In the last two decades, the application of flow cytometry analysis and molecular characterisation of different immune-related molecules have greatly improved our knowledge of the functional characterisation of haemocytes, underlying both common and distinct features of the immune system in different bivalve species (9, 148, 165, 221). Although granulocytes were largely suspected to play a prominent role in defence, few reports aimed to establish functional relationships between bivalve haemocyte subpopulations and immune capabilities (114, 170, 239). Therefore, this work attempts to gather information on different immune competencies among types of bivalve haemocytes.

#### 4.1.1 Phagocytosis and Encapsulation

One of the most important mechanisms of pathogen elimination in bivalves is phagocytosis, i.e. the engulfment of those foreign structures by haemocytes and their destruction (240). Phagocytosis of foreign structures by bivalve haemocytes was firstly reported in the oyster *C. gigas* (241). Studies on phagocytosis activity of bivalve haemocyte subpopulations have found diverse results. In the majority of bivalve species, both granulocytes and hyalinocytes are able to internalise foreign particles and pathogens; however, some studies have found that only granulocytes have phagocytic activity; this is the case of *C. edule* (77), *Tridacna crocea* (41), *M. galloprovincialis* (181), *C. virginica* (204), *P. nobilis* (179) and *P. fucata* (242). Nonetheless, other studies performed in some of the aforementioned species have pointed out that both haemocyte types have phagocytic ability as is indicated below.

Generally, granulocytes show higher phagocytosis capacities than hyalinocytes, which has been documented in oysters *C. ariakensis* (151), *C. gigas* (156), *C. hongkongensis* (157), *C. nippona* (160), *Crassostrea plicatula* (163), *C. virginica* (167), *Hyotissa hyotis* (148), *O. chilensis* (169), *O. edulis* (170), *O. circumpicta, S. kegaki* (148) and *S. glomerata* (173); in clams *M. lusoria* (61), *M. mercenaria* (66, 67), *R. decussatus* (72) and *R. philippinarum* (75); in the boreal tridonta *A. borealis* (40); in the geoduck clam *P. globosa* (45); in ark clams *A. broughtonii*, *A. kagoshimensis* and *T. granosa* (82); in the razor clam *Sinonovacula constricta* (92); in the cockle *C. glaucum* (79); in scallops *A. irradians* (93) and *A. farreri* (96); in marine mussels *B. azoricus* (101), *Mytilisepta virgata* (107), *M. chilensis* (109), *M. edulis* (110), *M. galloprovincialis* (113), *M. unguiculatus* (117), *P. canaliculus* (119), *P. perna* (120), *P. viridis* (122) and *X. securis* (124); and in freshwater mussels *Amblema plicata*, *Quadrula quadrula* (131), *D. chilensis* (128), *L. marginalis* (140) and *S. cumingii* (145) and in the pearl oyster *P. imbricata* (175). There are also studies where no differences in immune responses between the haemocytes types were found, as it happened in the ark clam *A. kagoshimensis* (86). In the clam *Callista chione* no differences in phagocytosis activity between granulocytes and hyalinocytes were observed (55).

Within granulocytes, eosinophilic granular haemocytes exhibited higher phagocytic activity than the basophilic ones in mussels, clams, cockles and oysters (23, 34, 72, 76, 78, 79, 102, 111, 163, 181, 243). Higher activity levels for phenoloxidases, peroxidases and superoxide dismutases, a greater production of superoxide radical (72, 111, 243), and a higher phagosome-lysosome fusion rate in eosinophilic granulocytes than in basophilic ones have been observed (76). Altogether suggest that eosinophilic granulocytes are more immune-reactive than basophilic granulocytes in bivalve immune defence (163).

Encapsulation is another major cellular immune defence process to eliminate foreign particles that are too large to be phagocytosed, by which haemocytes attach to the foreign organism to extracellularly destroy it (244). Different encapsulation activity was witnessed between haemolymph cells. In the oyster *C. virginica*, several studies reported the role of agranulocytes in encapsulation (245, 246). However, more recent studies propose granulocytes as the predominant cell types involved in such process in the oysters *S. glomerata* and *C. gigas* (156, 173).

Altogether it seems clear that in the vast majority of studied bivalves, granulocytes are the most active phagocytic cells. It has been hypothesised that the high phagocytosis activity of granulocytes is associated with the presence of granules with high levels of enzymatic activities which could act to kill and degrade the phagocytosed particles. Moreover, granulocytes had high ability to produce reactive oxygen species (ROS, radicals with microbicidal potential) and to form pseudopods. Finally, granulocytes contained more mitochondria, which could provide energy in the phagocytosis process to result in higher phagocytosis ability (75). However, some studies consider that hyalinocytes, as well as granulocytes, can be regarded as professional phagocytes (247), suggesting that they may target different types of microorganisms. While granulocytes possess a constantly high phagocytic index, the phagocytic index of hyalinocytes seemed related to the nature of the foreign material in the clam *R. decussatus* (72). The granulocytes of *C. gigas* were found to exhibit higher levels of phagocytic activity against bacteria and yeast than the agranulocytes, while the agranulocytes exhibited higher levels of phagocytosis against latex beads than granulocytes (248). In the same oyster species, hyalinocyte phagocytosis is regulated by an integrin-dependent mechanism, and it is thought that granulocytes have other receptors, still to be identified, to carry out this function (247). Different degradation pathways could be linked to different cell phagocytic abilities, depending on particle nature, to optimise degradation efficiency (127). Such observations suggest functional differences between haemocyte types and receptor-based initiation of phagocytosis (29).

#### 4.1.2 Enzymatic Lysosomal Content

Quantitative differences in the content of lysosomes between haemocyte types might be related to different cellular functions (148). The intracellular lysosomal enzyme contents of granulocytes have been reported much higher than those of hyalinocytes in several oyster species (148, 159, 169, 173), mussels (117, 122, 123, 181), clams (72, 74), ark clams (82), razor clams (92) and scallops (95). Granulocytes contain higher peroxidase, phenoloxidase and alkaline phosphatase activity than hyalinocytes in the scallop *A. farreri* (95). Granulocytes of the oyster *S. glomerata* are the haemocyte subpopulation with greater levels of acid phosphatase and phenoloxidase enzymatic activities (173). Acid phosphatase activity have been found only in granulocytes in the boreal tridonta *A. borealis* (40). In the pen-shell *P. nobilis*, granulocytes were more positive to hydrolases than hyalinocytes (179). These results indicate the important role played by granulocytes in immune reactions.

On the contrary, agranulocytes (large and small hyalinocytes) seemed more diverse in protein content than the granulocytes in the oyster *O. edulis* (249). In the freshwater mussel *D. polymorpha*, presence of acid phosphatase and non−specific esterase was detected in both hyalinocytes and granulocytes, while ß−glucuronidase was detected only in hyalinocytes (126). The differential distribution of hydrolytic enzymes between granulocytes and hyalinocytes is associated with different physiological and immune responses (74).

#### 4.1.3 Oxidative Activity

The production of radicals with microbicidal activity, such as reactive oxygen species (ROS) and reactive nitrogen species (RNS), is induced after phagocytosis and it may be used to evaluate the immunocompetence in different haemocyte subsets (156). Oxidative activity through ROS production is generally higher in granulocytes than in hyalinocytes, which was observed in oysters *C. ariakensis* (151), *C. gigas* (154, 156, 250, 251), *C. hongkongensis* (157), *C. nippona* (160), *C. virginica* (167, 252), *O. chilensis* (169), *Hyotissa hyotis*, *Ostrea circumpicta*, *S. kegaki (* 148 *)*, and *S. glomerata* (173); in clams *R. decussatus* (73) and *R. philippinarum* (75); in the geoduck clam *P. globosa* (45); in ark clams *A. broughtonii*, *A. kagoshimensis* and *T. granosa* (82); in marine mussels *M. galloprovincialis* (114), *M. unguiculatus* (117), *P. canaliculus* (119), *P. viridis* (122), and *X. securis* (124); and in freshwater mussels *L. marginalis* (140). In *L. marginalis*, granulocytes were identified as the principal phagocytes with prominent activity of superoxide anion (a ROS) and nitric oxide (NO, a RNS) (140). The higher ROS production of granulocytes may be also related to a more active metabolism (253). Moreover, it has been shown that the oxidative process within haemocytes plays a key role in the formation of extracellular DNA traps in oysters (254), clams (255, 256) and mussels (257). These extracellular traps (ETs) carrying AMPs and hydrolases released from granules could surround, entangle and eventually kill the pathogens, operating as antimicrobial effectors during the innate immune response. Thus, in marine bivalves, ETs participate in host defence by capturing large numbers of microbes and preventing their dissemination (254, 258, 259). Differences in ROS production between bivalve haemocyte subpopulations could be related with differences on such immune responses (158); in higher organisms granulocytes seem to be more implicated in ETs formation (260).

Other studies showed that granulocytes and hyalinocytes have the ability to produce different ROS and RNS (148). In the freshwater mussel *D. polymorpha*, hyalinocytes showed the highest intracellular ROS production (127). Oyster *C. gigas* hyalinocytes produced more reactive nitrogen species (RNS) than granulocytes (250). These results could indicate that the main haemocyte types have different capabilities for ROS/RNS production response. Both cell types possess NADPH-oxidase and NO-synthase-like pathways to produce ROS/RNS but the NO-synthase pathway seemed more dominant in hyalinocytes, whereas NADPH-oxidase was more active in granulocytes (250). Thus, differences in oxidative activity between the granulocytes and hyalinocytes could be associated with the differential involvement of ROS production pathways of the two haemocyte types. Moreover, regulation of phagocytosis of diverse targets and regulation of ROS and NO production, reveals haemocyte type-specific variations in signalling mechanisms, which could be due to the differential expression of membrane receptors (114). These observations led to hypothesise that mechanisms for killing foreign particles might be different between hyalinocytes and granulocytes (204). It was also suggested that differences in ROS production between haemocyte types may be associated with the functional role and the morphological structure of each cell type (261), being granulocytes more oxygen demanding than agranular cells, because the former type possess more complicated ultrastructure with numerous mitochondria and endoplasmic reticulum (60).

#### 4.1.4 Aggregation and Wound Healing

Even though the hyalinocytes are not as avidly phagocytic as granulocytes, it is believed that they play a central role in haemocyte aggregation processes and wound healing (20, 173, 240, 262, 263). In the giant clam *T. crocea*, hyalinocytes were located in the core of haemocyte aggregations associated with wound healing (41). In the oyster *S. glomerata*, hyalinocytes were shown to have a central role in haemocyte aggregation processes (173). Similar results have been observed in the clam *R. philippinarum* (75). In the mussel *P. viridis*, hyalinocytes were shown to have a central role in either haemocyte aggregation or coagulation processes (123).

#### 4.1.5 Apoptosis

Granulocytes normally show higher levels of apoptosis than hyalinocytes, possibly due to increased phagocytic activity and respiratory burst (264, 265). Differences in mortality have been found between both cell types, being greater in granulocytes; such difference in mortality may be related to the difference in ROS production (266). In mussels *M. galloprovincialis*, apoptotic levels seemed to be higher in granulocytes (267). Similarly, apoptosis after pesticides exposure have been found higher in granulocytes than in agranulocytes in the freshwater mussel *L. marginalis* (268). In the mussel *M. galloprovincialis*, although both haemocytes subpopulations were susceptible to UV light treatment, the damages induced in hyalinocytes were detected earlier than in granulocytes, which led authors to suggest that the cytoplasmic granules of granulocytes could have some protective effect against apoptosis induced by UV radiation (269). On the contrary, in the oyster *O. edulis*, granulocytes appeared more affected by apoptosis than hyalinocytes (270); after a proteomic approach more proteins related with apoptosis were identified in granulocytes (237, 238). In the clam *R. decussatus*, haemocytes stimulation with lipopolysaccharide induced a significant up-regulation of a gene with important roles in the apoptotic process, with a maximum level registered in granulocytes (73). A recent study suggested that apoptosis of *C. virginica* granulocytes may be Apoptosis protein inhibitor (IAP)-dependent and involve caspase-independent pathways (271).

#### 4.1.6 Immune Molecules and Pathways

Higher content of molecules involved in immune response and higher expression levels of various immune related genes in the granulocytes of the oyster *C. gigas* compared to semigranulocytes and agranulocytes led to suggest that the granulocytes are the main immunocompetent haemocytes (156). Previous studies have shown that defensins and mytilins are stored mainly in granulocytes (272, 273). Myticin C was found expressed in granulocytes of mussels *M. galloprovincialis* (274); on the contrary, Myticin C was identified expressed in hyalinocytes and not in granulocytes in the oyster *O. edulis* (238). Haemolymph cells exhibited distinct inter-specific lectin binding in clams (275), marine mussels (101, 102, 276) and freshwater mussels (132), suggesting that haemocytes subpopulations may express different sugar moieties and perform disparate functions. Oyster *C. virginica* galectins CvGal1 and CvGal2 and clam *R. philippinarum* galectin MaGal1 were identified with strong ability to recognise parasites of the genus *Perkinsus* (277–279); one of them, CvGal1, has been observed to be secreted by granulocytes (277). In oysters *O. edulis*, the lectins galectin-4 isoform X1 and ß-1,4-N-acetylgalactosaminyltransferase bre-4-like appear to be expressed in hyalinocytes (238). Lectin-like receptors (LLRs) were found to play important roles in the phagocytosis of granulocytes and semigranulocytes in *C. gigas* (280). In the same way, antimicrobial peptides were found within granular haemocytes in the scallop *N. subnodosus* (97). ATP-binding cassette (ABC) proteins associated with the multixenobiotic resistance were observed expressed differently between haemocyte types in mussels and oysters (281, 282). Cathepsin L gene expression, involved in the inflammatory response, was strongly associated with the number of circulating granulocytes in *C. gigas* (283). SPRY (sp1A/ryanodine receptor) domain-containing SOCS box protein (CgSPSB), which play an important role in the regulation of cytokine production in *C. gigas*, was found mainly distributed in the cytoplasm of granulocytes (284). In the same species, immune related genes, including CgTLR, CgClathrin, CgATPeV, CgLysozyme, CgDefensin and CgIL-17, were mainly expressed in granulocytes (156). CgCaspase-8-2 was found mainly distributed in granulocytes. This protein functioned as important protease to be involved in the anti-bacterial immunity responses through inducing the expressions of cytokines, defensin and autophagy-related genes (285). Aminopeptidase was found expressed in eosinophilic granulocytes from *M. edulis* (286). Chitinase (Cg-Chit) seems to play redundant functions for the immune responses in *C. gigas* and it is specifically expressed in granulocytes (287). Alcohol acyltransferase (CgAATase), enzyme involved in immune response, was found to be mainly expressed in granulocytes (288). Glutamic acid decarboxylase (CgGAD), enzyme responsible to catalyse the production of gamma aminobutyric acid (GABA), an important neurotransmitter of the GABAergic system, was dominantly expressed in granulocytes of *C. gigas* (289). In the same species, dopamine β-hydroxylase (DBH), a norepinephrine synthesising enzyme, was highly expressed in granulocytes and involved in neuroendocrine and immune response (290). Differences in the expression of neuroendocrine immunomodulation (NEI) related proteins between oyster *O. edulis* haemocyte types have been detected (238). Some antigens reacted differently in granulocytes and agranulocytes in the deep-sea symbiotic mussel *B. japonicus* (291).

Different proteins with key roles in important immune pathways were identified in the main haemocyte types of the oyster *O. edulis*, with more proteins involved in the MAPK, Ras and NF-κβ pathways in granulocytes, while in hyalinocytes there were more identified proteins that participate in the Wnt signalling pathway (238). In the oyster *C. gigas*, the MAPK pathway was found to participate in granulocytes to regulate functional activities prior than in hyalinocytes, also suggesting functional differentiation of haemocyte types (216). A recent study proposed that oyster *C. hongkongensis* granulocytes mainly participate in immune response through the NF-κB pathway and autophagy process (182).

### 4.2 Other Physiological Processes

Differences in cellular metabolism suggest, as discussed above, that granulocytes and hyalinocytes may be involved in different physiological functions.

#### 4.2.1 Motility

Motility is crucial for haemocytes to carry out multiple functions, including immune response. Previous reports showed variations related to the locomotion and cytoskeleton properties among haemocyte types of bivalves. In the pearl oyster *P. imbricata*, granulocytes showed amoeboid locomotion and directional, while hyalinocytes appeared to be less mobile, often adhered to a substrate and spread multidirectionally (175). However, hyalinocytes also showed the ability for amoeboid movement in the oyster *C. hongkongensis* (157). Motility dynamics (either ameboid or based on pseudopod formation) has been emphasised as an important criterion for morphofunctional classification of mussel *M. edulis* haemocytes (112).

#### 4.2.2 Spawning

Spawning in marine bivalves is a great energy-demanding process, and it often results in lethal and sublethal stresses during the post-spawning period, including depressed immune capacity (292). Female and male clams *R. philippinarum* showed different haemocyte populations during the pre-spawning phase, females having a higher fraction of granulocytes and males of hyalinocytes (188). Granulocyte percentage was higher in spring and early summer in the scallop *A. farreri*, and lower in summer and early autumn, the period corresponding to reproduction completion (293). The phagocytosis capacities of haemocytes were significantly reduced during the post-spawning period in the oyster *S. kegaki*, being more pronounced in granulocytes (190). In the oyster *C. hongkongensis*, males exhibited a more powerful cellular immune response than females after spawning, the former showing higher esterase activities, lysosomal masses, nitric oxide levels, and granulocyte numbers (294). Spawning was shown as a stressful activity inducing depressed immunological capacities in the ark clam *T. granosa*, with dramatical decline of granulocyte phagocytosis capacity in individuals engaged in active spawning while the production of ROS (indicative of stress) of the granulocytes and the erythrocytes type II increased linearly during the post-spawning period (292).

#### 4.2.3 Shell Formation and Repair

Regarding shell regeneration, granulocytes have been shown to participate in the synthesis and transportation of CaCO_3_ in oysters *C. virginica* (25, 295) and *C. gigas* (296, 297), as well as in the deep-sea vent mussel *B. azoricus* (100, 298) and in pearl oysters *P. fucata* (242, 299). In the oyster *C. gigas*, several shell formation-related genes are highly expressed in H2 and H3 haemocytes (similar to granulocytes), thus these haemocyte types are potential players in biomineralisation processes (300). Moreover, the direct involvement of granulocytes in the formation of the prismatic layer has been found in the pearl oyster *P. fucata* (299). In the same species, Huang *et al.* (2018) successfully identified numerous calcium-rich vesicles and crystals in granulocytes and assigned to this cell type the strongest ability of migration (242). Altogether, the granulocytes may be a calcium pool and act as a calcium conveyor during shell formation and may participate in the initiating process of bivalve shell mineralisation (301).

## 5 Differential Response to Stress

Bivalve molluscs are poikilotherm organisms and multiple physiological functions of bivalves show seasonal variation. Consistently, as haemocytes are involved in multiple functions, total haemocyte count and the relative abundance of each haemocyte type in the haemolymph of bivalves also show seasonal variation (161, 302–304), which has to be considered normal. Additionally, the internal responses of bivalves to invasive pathogens, natural environmental impacts, and pollutants are mediated at least in part by haemocytes (23, 39). Furthermore, any effect that environmental stressors exert on the bivalve haemocyte proportions or functioning may ultimately result in a reduction of immune response effectivity, whereas bivalves are able to overcome well exposure to a wide variety of pathogens if the immune system is not over-challenged (147). Changes in total haemocyte count and the relative proportions of granular and agranular cells often are used as indices of bivalve immune status (30, 31, 248). There are many studies focused on the haemocyte immune response to a wide range of stressors in which it is possible to suggest functional differences between haemolymph cell types. Research literature focusing on effects of biotic and abiotic stress on bivalve haemocyte subpopulations is summarised in **Tables 2** and **3**.

**Table 2:** Literature survey of reported effects of biotic stress on bivalve haemocyte subpopulations.

Species	Type of pathogen or disease							Effect	References
	Juvenile oyster disease	Bonamiosis	Perkinsosis	Marteliosis	Bacteria	Trematodes	Copepods		
*Anadara trapezia*						X		≠ G/H ratio	(87)
*Cerastoderma edule*						X		Recruiting G	(305)
*Azumapecten farreri*					X			**↓** G	(306)
*Crassostrea brasiliana*			X					≠ G/H ratio	(149)
*Crassostrea gigas*					X			**↑** ROS H **↑↑** ROS G	(307)
					X			≠ G/SemiG/H ratio	(308)
*Crassostrea madrasensis*					X			**↓** H **↑** G	(309)
*Crassostrea virginica*	X							**↑** % G	(31)
			X					**↓** G apoptosis	(271)
*Mya arenaria*					X			≠ G/H ratio	(310)
*Mytilus galloprovincialis*				X				**↓** % G	(311)
							X	**↓** % G	(185)
*Ostrea edulis*					X			**↑** G	(312)
		X						**↑** sH **↓** G	(171, 313)
*Perna perna*						X		**↓** % G	(314)
*Ruditapes philippinarum*					X			**↓** H **↑** G	(315)
					X			**↓** G	(316)
			X					**↑** G	(303)
*Sinohyriopsis cumingii*					X			**↑** G	(146)
**Species** **(resistant or tolerant selected stocks)**	**Type of pathogen or disease**							**Effect**	**References**
	**MSX disease**		**Perkinsosis**	**Bacteria**		**Bonamiosis**	**Marteliosis**		
*Crassostrea virginica*	X							**↑** % G	(317)
			X					**↑** % G **↑** [lysozyme]	(318)
*Ostrea chilensis*						X		H more infected	(319)
*Ostrea edulis*						X		**↑** % G	(313, 320)
						X		Multiply more in H	(321)
*Ruditapes philippinarum*				X				**↑** G **↑**Phagocytosis	(191)
*Saccostrea glomerata*							X	**↑** G **↑**Phagocytosis	(322)
							X	**↑** G **↑** ROS	(174)
**Species**	**HAB**							**Effect**	**References**
*Argopecten irradians irradians*	X							1° **↑** H **↓** G 2° **↓** H **↑** G	(323)
*Crassostrea gigas*	X							**↑** G	(324)
	X							**↑** G size	(325)
	X							≠ G/H ratio	(326)
	Saxitoxin							H more susceptible	(327)
*Crassostrea virginica*	X							**↑** G **↑**Phagocytosis	(328)
*Dreissena polymorpha*	Cyanotoxin*							**↑** G	(329)
*Mytilus chilensis*	Saxitoxin							Affect more G	(109)
*Mytilus edulis*	X							1° **↓** G 2° **↑** G	(330)
	X							**↓** G	(331)
*Mytilus galloprovincialis*	X							**↓** G	(332)
	X							≠ G/H ratio	(333)
*Perna perna*	X							**↓** G	(334)
*Ruditapes philippinarum*	X							**↑** G	(335)

*Cyanotoxin is produced by bacteria, not by HABs.

G, granulocyte; sG, small granulocyte; SemiG, semigranulocyte; H, hyalinocyte; sH, small hyalinocyte; HAB, harmful algal bloom.

**Table 3:** Literature survey of reported effects of abiotic stress on bivalve haemocyte subpopulations.

Species	Type of stressor													Effect	Reference
	Temperature increase		Acidification		Salinity		Hypoxia		Heavy Metals		POPs				
*Azumapecten farreri*	X													**↓** % G	(293)
														**↓** G	(306)
*Crassostrea gigas*	X						X							**↓** G mortality **↓** ROS	(266)
														**↑** A_G_ **↓** ROS G	(261, 336)
*Crassostrea virginica*	X													**↑** % G	(30)
	X													**↑** H mortality	(252)
*Mytilus coruscus*			X				X							≠ G/H ratio	(337)
*Mytilus edulis*			X											↑ G damage ↓Phagocytosis	(338)
*Mytilus galloprovincialis*	X		X				X		X					**↓** G	(339)
							X							**↑** G **↓** ROS G **↓** A_G_	(115)
*Pinctada fucata*	X		X											**↑** lH **↑** G	(176)
*Pinctada imbricata*					**↓**									**↑** G	(340)
*Ruditapes philippinarum*					**↑**									**↓** % G	(341)
	X													**↑** G	(303)
*Spisula solidissima*	X													**↓** % G	(18)
*Tegillarca granosa*			X											**↓** % rG	(342)
			X								X			**↓** % rG **↑** bG **↓**Phagocytosis	(343)
*Unio pictorum*	X													**↑**H mortality	(147)
**Species**	**Type of pollution**													**Effect**	**Reference**
	**Crude oil**	**Pharmaceutical contaminants**	**Insecticides**	**Cigarette butts**	**PCBs**	**Trace Metals**	**Cadmium (Cd)**	**Copper (Cu)**	**Zinc (Zn)**	**Xenobiotics**	**POPs**	**Microplastics (MPs)**	**Nanoplastics (NPs)**		
*Anodontites trapesiali*				X										**↑** G	(129)
*Azumapecten farreri*					X									**↓** G **↓**Phagocytosis	(344)
*Crassostrea gigas*	X													**↓** G	(155)
										X				**↑** % G	(345)
*Crassostrea hongkongensis*									X					**↑** G mortality **↓** G **↑** A_G_ **↑**semiG G + sensitive	(158)
*Dreissena polymorpha*							X							H + sensitive	(346)
*Mytilus edulis*	X													1° **↓** G 2° **↑** G	(347)
								X						↓ eG ↑ bG	(348)
												X		Granulocytoma	(349)
											X			**↓** G	(350)
*Mytilus galloprovincialis*		X												**↑** G/H ratio	(351)
		X												**↓** G **↓** G/H ratio	(352)
							X							**↑** G/H ratio	(353)
												X		**↓** G/H ratio	(354)
												X		**↑** G	(355)
													X	↓ bG **↑** H	(356)
													X	G + sensitive	(357)
*Mytilus* spp.												X	X	≠ G/H ratio	(358)
												X		**↓** G	(359)
*Perna canaliculus*							X							**↑** G	(118)
						X								**↓** H	(360)
*Ruditapes decussatus*								X						**↑** H Phagocytosis	(192)
*Ruditapes philippinarum*	X													**↑** G	(361)
*Saccostrea glomerata*			X											**↓** G aggregation	(362)
*Tegillarca granosa*							X							**↓** rG	(363)
													X	**↓** rG **↓**Phagocytosis	(364–366)
					X									**↓** rG **↑** bG	(367)
												X		**↓** rG **↓**Phagocytosis	(368)
												X		**↓** rG **↑** bG	(369)

G, granulocyte; sG, small granulocyte; A_G_, agranulocyte; rG, red granulocyte; eG, eosinophil granulocyte; bG, basophil granulocyte; H, hyalinocyte; lH, large hyalinocyte; sH, small hyalinocyte; PCBs, polychlorinated biphenyls; POPs, persistent organic pollutants.

### 5.1 Biotic Stress: Pathogens and Harmful Algal Blooms

The relative abundance of each cell type in the haemolymph of bivalve molluscs is variable and is influenced by the presence of certain pathogens (321). Regarding the reaction against bacteria, juvenile oyster disease was correlated with altered cell ratios resulting in an increase in the percentage of granulocytes in oysters *C. virginica* (31). Clams *R. philippinarum* challenged with various *Vibrio* species showed significant decrease of hyalinocytes and increase of granulocytes, suggesting different involvement of each haemocyte type in antibacterial defence (315). On the contrary, the granulocyte concentration decreased in the same clam species after infection with *Vibrio tapetis* (316). In the oyster *C. gigas*, strong enhancement of haemocyte ROS production following bacterial infection was observed, which was higher in granulocytes than hyalinocytes (307). In the same species, modifications of the proportion of the haemolymph cells have been observed after bacterial challenge (308). Granulocytes were reported as the main haemocyte type involved in antibacterial response in the freshwater mussel *A. cygnea* (370). In the marine mussel *M. galloprovincialis*, the haemocyte types exhibited distinct responses to infection by various bacterial species of the genera *Vibrio* and *Micrococcus* (221, 371). The infection with bacteria *Listonella anguilarum* also induced changes in the relative abundance of haemocyte types of the oyster *O. edulis* haemolymph, favouring granulocytes (312). In the clam *Mya arenaria*, independent modifications after bacterial infection were observed in the proportions of haemocyte subpopulations established by their lysosomal content, suggesting specific modulation of bivalve responses against pathogenic bacteria that would include degranulation (310). Bacterial challenge produced an increase in the percentage of granulocytes and a decrease in that of hyalinocytes in the oyster *C. madrasensis*, suggesting the main involvement of granulocytes in immune response (309). Similar results were found in the freshwater mussel *S. cumingii*, with total haemocyte count increase, especially the proportion of granulocytes, after bacterial infection (146). On the contrary, granulocyte count decreased in the scallop *A. farreri* when challenged with bacteria; such decline increased at high temperature (306). Regarding metazoan pathogens, it has been suggested that the bivalve immune system responds to trematode invasion by recruiting granulocytes (305). Thus, in the mussel *P. perna* a decrease in the percentage of granulocytes in the circulating haemolymph was observed associated with trematode infection (314). Trematodes also caused change in the proportions of the haemocyte types in the ark clam *A. trapezia* (87). A decrease in the percentage of granulocytes upon infestation by copepods was found in the mussel *M. galloprovincialis*, which was interpreted as a tendency for invertebrate haemocytes to degranulate in response to parasitism (185). Regarding responses to protistan pathogens, *Perkinsus* spp. appear to induce changes in the proportions of haemocyte types in the oyster *C. brasiliana* (149), suppression of granulocyte apoptosis in *C. virginica* (271) and a significant increase in granulocyte concentration in clams *R. philippinarum* (303). In the oyster *O. edulis*, increase of the proportion of small hyalinocytes and decrease of that of granulocytes associated with *Bonamia ostreae* infection was observed (171, 313). Hyalinocytes were abundant in tissues heavily infected with *Haplosporidium* sp. in the oyster *S. cucullata* (172). In the mussel *M. galloprovincialis*, lower percentage of granulocytes was observed in the presence of *Marteilia* spp. parasites (311).

A more precise perspective on the relevance of the haemocyte types in the response against pathogens can be attained from comparison between resistant (or tolerant) and susceptible hosts (372). Oysters *C. virginica* resistant to the protistan *Haplosporidium nelsoni* showed significantly higher percentage of granulocytes (317). In the same species, higher percentage of granulocytes was found in the haemolymph of oysters with higher tolerance to infection with the protistan *Perkinsus marinus* (318). Similarly, clams *R. philippinarum* resistant to *Vibrio tapetis* had relatively more granular haemocytes, resulting in increased phagocytic capacity (191). Oysters *O. edulis* with increased resistance to *B. ostreae* showed different haemocyte counts than susceptible ones (320), displaying higher percentage of granulocytes (313). Remarkably, the intracellular parasite *B. ostreae* multiplies more successfully within hyalinocytes than granulocytes (321); similarly, hyalinocytes of the oyster *O. chilensis* are known to be preferentially infected by *Bonamia exitiosa* (319). In oysters *S. glomerata*, resistance to the protistan *Marteilia sydneyi* is linked to increased frequencies of granulocytes, likely due to higher phagocytic activity and higher levels of phenoloxidase in this haemocyte type (322) and higher ROS production (174). A positive health effect of treatment of *C. gigas* with brown and red seaweeds has been suggested; treated oysters showed significant increase in granulocyte count and a low pathogen prevalence (373).

Several studies have reported modulation of bivalve haemocyte variables in response to harmful algal bloom (HAB) exposure (374). Exposure to toxin-producing dinoflagellates increased oyster *C. virginica* haemocyte phagocytosis ability and granulocyte subpopulation (328). In the case of the scallop *A. irradians*, a biphasic effect was detected, with initial increase of hyalinocyte count and decrease of that of granulocytes and posterior hyalinocyte decrease and increase of granulocyte count (323). Variation of the differential haemocyte count was also detected in the mussel *M. edulis*, in which eosinophilic granulocytes decreased at the beginning of exposure but increased after a few days (330). In the same mussel species, degranulation of eosinophilic granulocytes associated with exposure to toxin-producing dinoflagellates was reported (331). Other reported effects of exposure to toxin-producing dinoflagellates were drastic increase of granulocytes (324), increase of granulocyte size (325), and changes in the haemocyte type relative abundance in the oyster *C. gigas* (326); doubling number of granulocytes in clams *R. philippinarum* (335); decrease in the percentage of granulocytes in the mussel *P. perna* (334); and anomalous decrement of granulocytes (332) and variations of the granulocytes/hyalinocytes (G/H) ratio in the mussel *M. galloprovincialis* (333). *In vitro* exposure to saxitoxin, a neurotoxin produced by dinoflagellates, affected more granulocytes than hyalinocytes of the mussel *M. chilensis* (109), while oyster *C. gigas* hyalinocytes were found to be highly responsive (327). Exposure to cyanotoxin producing bacteria increased the relative proportion of granulocytes in the freshwater mussel *D. polymorpha* (329).

### 5.2 Abiotic Stress: Temperature, Salinity, Acidification, Hypoxia and Pollution

High water temperature can influence haemocyte parameters in bivalves, including haemocyte number, motility, viability, adhesive capacity, phagocytic ability, membrane permeability, and intracellular enzyme activities, which may result in weakened ability to mount an immune defence (375, 376). Temperature increase was associated with higher percentage of granulocytes in the oyster *C. virginica* (30) and higher mortality of hyalinocytes (252). A positive correlation between temperature and granulocyte counts was observed in the clam *R. philippinarum* (303). High values of granulocyte percentage were observed in the scallop *A. farreri* during the period of favourable water temperature, whereas low values were found during the period of high water temperature (293). Increase of the percentage of large hyalinocytes and granulocytes in the pearl oyster *P. fucata* was proposed to be associated with warming and ocean acidification (176). On the contrary, increasing temperature was found associated with decrease of the percentage of granulocytes in the clam *Spisula solidissima* (18). Hyalinocyte mortality was significantly increased at high temperature while no effect of temperature was evident in the granulocyte mortality of the freshwater mussel *Unio pictorum* (147). The phagocytic capacity of granulocytes from the mussels *M. virgata* under the heatwave condition decreased to one-third of the values in control mussels (107). Regarding salinity effects, decrease of the number of granulocytes in the clam *R. philippinarum* was reported as consequence of salinity increment (341). Similarly, the frequency of granulocytes increased significantly when pearl oysters *P. imbricata* were stressed by hypo-saline conditions (340). In the oyster *Crassostrea corteziensis*, hyalinocyte and granulocyte counts have higher values in hyposaline stress conditions and lower values in hypersaline stress conditions; however, these haemocyte type counts change at a different rate (377). Realistic pH reduction, as expected with ocean acidification, induced a decrease of the percentage of red granulocytes in the ark clam *T. granosa* (342) and an increment of the ratio of damaged granulocytes in mussels *M. edulis* (338). Mussels *M. galloprovincialis* co-exposed to high temperature, acidification and cadmium experienced significantly reduction of the granulocyte proportion (339). Changes of the three subpopulations of the mussel *M. coruscus* under a short-term exposure to acidification and hypoxia have been observed (337). Hypoxia also induced substantial increase of granulocytes and a decrease of agranulocytes and intracellular ROS production in granulocytes in the mussel *M. galloprovincialis* (115). However, in the oyster *C. gigas*, hypoxia influenced agranular and granular cells differently, with a higher decrease of ROS production in granulocytes and an increase of agranulocytes number (261, 336). In the same species, O_2_ deprivation resulted in a strong decrease of granulocyte mortality potentially linked with a decrease of ROS production (266).

Bivalves have been widely used as sentinel organisms in the biomonitoring of aquatic pollution (7). Pollution may result in the death of haemocytes owing to lysis and in changes in the proportions of their main cell types; this fact had been observed in mussels, oysters and clams (155, 348, 378). Moreover, the formation of granulocytomas is an inflammatory cellular response associated with environmental pollution. Granulocytomas are a bioindicator of the haemocytic response to pollutants as well as a general loss of health in bivalves (379, 380). Thus, morphometric alterations of granulocytes may be used in a biomarker battery in aquatic environmental monitoring (381). The clam *M. arenaria* collected from polluted sediments had a higher proportion of granulocytes compared to those from a relatively clean area, indicating possible haemocyte involvement in sequestration of chemical pollutants (382). Also in the mussel *M. galloprovincialis*, the numbers of eosinophilic and basophilic granulocytes were higher in polluted than in clean areas (383). In the mussel *M. kurilensis*, pollution resulted in significantly decrease of the percentage of agranulocytes and phagocytic activity and the formation of granulocytomas (35). In mussels *M. edulis* exposed to crude oil, initial reduction of granulocytes followed by granulocyte increase was observed, which was considered an adaptive response to stress (347). One year after a disastrous oil spill in Korea, decreased granulocyte count was reported in the oyster *C. gigas* (155), while higher proportion of granulocytes in the clam *R. philippinarum* was reported two years after the spill (361). Different types of pharmaceutical contaminants produce variation in the mussel *M. galloprovincialis* physiological response, particularly inducing changes of the G/H ratio, some compounds increasing (351), while decreasing it others (352). Insecticides also impair bivalve physiology and, in the oyster *S*. *glomerata*, granulocytes and hyalinocytes respond differently to different concentrations of pesticides (362). Pollution by leachates from cigarette butts induced increase of granulocyte proportion in the freshwater mussel *A. trapesiali* (129). A variety of responses in differential haemocyte count have been previously reported in bivalves upon exposure to metal toxicants. The proportion of red granulocytes were significantly reduced after 10 days exposure of the ark clam *T. granosa* to Cd^2+^ spiked seawater, which suggested significant immunotoxicity of Cd^2+^ to this species (363). In mussels *M. edulis*, decrease of the proportion of circulating eosinophilic granulocytes and increase of basophils after copper exposure was observed (348). In mussels *M. galloprovincialis*, granular haemocytes were found less sensitive to genotoxic damage compared with agranular haemocytes (384). Exposure to Cd increased the proportion of granulocytes in the mussel *P. canaliculus* (118) and the G/H ratio in *M. galloprovincialis* (353). Also in the mussel *P. canaliculus*, metal pollution induced hyalinocyte decrease (360). In freshwater mussels *D. polymorpha*, the different response of haemocyte types to Cd led to consider granulocytes with higher capacity to regulate oxidative stress and greater involvement in essential metal transport or sequestration of heavy metals (346). In the clam *R. decussatus*, higher phagocytic activity in hyalinocytes than in granulocytes was reported when the cells were *in vitro* exposed to CuSO_4_ (192). Zinc-contaminated oysters *C. hongkongensis* showed increase of granulocyte mortality, which suggested that granulocytes were the most sensitive cell type in responding to Zn; moreover, the granulocyte number decreased whereas those of semigranulocytes and agranulocytes increased (158). In the same species, specific responses of granulocyte, semigranulocyte and hyalinocyte have been detected to copper; granulocyte was the most important responsive cell type and displayed heterogeneity responses of its two distinguished subtypes (385). In the oyster *C. gigas*, granulocyte percentage increased in the presence of hydrocarbons, which led authors to hypothesise that granulocytes may be more resistant than hyalinocytes (345). The proportion of granulocytes and phagocytosis ability decreased while the proportion of hyalinocytes increased in the scallop *A. farreri* after exposure to polychlorinated biphenyls (PCBs) (344). In the ark clam *T. granosa*, exposure to high doses of PCB led to red granulocyte percentage decrease and basophil granulocyte increase (367). Nanoplastics (NPs) and microplastics (MPs) had a significant effect on G/H ratios of *Mytilus* spp. (356, 358, 359), with a decrease of granulocyte concentrations by MPs (359), a decrease in basophil granulocytes and an increase in hyalinocytes in *M. galloprovincialis* by NPs (356). Exposure of mussels *M. galloprovincialis* to benzo[a]pyrene (B[a]P) and MPs increased granulocytes proportion (355), while, in another study, MPs induced decrease of the G/H ratio (354). In mussels *M. edulis*, MPs caused the formation of granulocytomas, an inflammatory response mainly due to eosinophilic granulocytes (349). Exposure to NPs causes different immune responses between haemocyte subpopulations. In fact, granulocytes of the mussel *M. galloprovincialis* appeared to be more sensitive than hyalinocytes (357). Ark clams *T. granosa* showed phagocytic activity and red granulocytes ratio significantly reduced after exposure to NPs (364–366) and B[a]P (368). Similarly, exposure to MPs and polycyclic aromatic hydrocarbons (PAHs) led to a significant decrease of the proportion of red granulocytes and increase of basophil granulocytes in *T. granosa*. Moreover, the ark clams coexposed to MPs and PAHs showed significantly lower proportion of red granulocytes and higher of basophil granulocytes than clams exposed to MPs or PAHs alone (369). In the same species, a significant decrease detected in phagocytosis when exposed to B[a]P under low pH simulating future ocean acidification scenarios may be attributed to significant reduction of red granulocyte count (343). Long-time-exposure to persistent organic pollutants (POPs) in mussels *M. edulis* may decrease the proportion of granulocytes, suggesting that such haemocyte type may be more sensitive to these pollutants (350).

Considering the important effects of biotic and abiotic stressors on the bivalve haemolymph cells, extensive monitoring studies of the morphofunctional properties of the haemocytes of bivalves in the natural environment would be useful to state reliable criteria for diagnosing the physiological status of bivalves (34).

## 6 Conclusion

Due to bivalve mollusc diversity and functional heterogeneity, haemocyte types vary from species to species and, nowadays, there is not a unified nomenclature that applies to all bivalves. Moreover, functions of each haemocyte type cannot be reliably extrapolated among all species. Haemocyte subpopulations own distinct properties that should be considered when characterising the overall immune related functions of bivalves. Granulocytes and hyalinocytes display differences in their metabolism and immune abilities, which implies that they play different physiological and immunological roles that should be deeply explored. Infections induce changes in the proportions of haemocyte types, which points out differential involvement of the haemocyte types in immune response. Abiotic stressors also alter the relative abundance of haemocyte types, which highlights functional differences. The ratio of cell types in the haemocyte community could be noted as indicator of immune function, being an important immune parameter to assess the bivalve health-status.

## Author Contributions

NB: conceptualisation, methodology, literature review, and writing – original draft. FM: conceptualisation, literature review, writing – review and editing. AC: writing - review and editing. AV: conceptualisation, literature review, writing – review and editing, and supervision. All authors contributed to the article and approved the submitted version.

## Conflict of Interest

The authors declare that the research was conducted in the absence of any commercial or financial relationships that could be construed as a potential conflict of interest.

## Publisher’s Note

All claims expressed in this article are solely those of the authors and do not necessarily represent those of their affiliated organizations, or those of the publisher, the editors and the reviewers. Any product that may be evaluated in this article, or claim that may be made by its manufacturer, is not guaranteed or endorsed by the publisher.
